# Impact of network centrality and income on slowing infection spread after outbreaks

**DOI:** 10.1007/s41109-023-00540-z

**Published:** 2023-02-24

**Authors:** Shiv G. Yücel, Rafael H. M. Pereira, Pedro S. Peixoto, Chico Q. Camargo

**Affiliations:** 1 School of Geography and the Environment, University of Oxford, Oxford, UK; 2 Institute for Applied Economic Research (IPEA), Brasília, Brazil; 3 Applied Mathematics Department, University of São Paulo, São Paulo, Brazil; 4 Department of Computer Science, University of Exeter, Exeter, UK

**Keywords:** Human mobility, Socio-economic inequality, Epidemic intervention effectiveness, Spatial analysis

## Abstract

The COVID-19 pandemic has shed light on how the spread of infectious diseases worldwide are importantly shaped by both human mobility networks and socio-economic factors. However, few studies look at how both socio-economic conditions and the complex network properties of human mobility patterns interact, and how they influence outbreaks together. We introduce a novel methodology, called the Infection Delay Model, to calculate how the arrival time of an infection varies geographically, considering both effective distance-based metrics and differences in regions’ capacity to isolate—a feature associated with socio-economic inequalities. To illustrate an application of the Infection Delay Model, this paper integrates household travel survey data with cell phone mobility data from the São Paulo metropolitan region to assess the effectiveness of lockdowns to slow the spread of COVID-19. Rather than operating under the assumption that the next pandemic will begin in the same region as the last, the model estimates infection delays under every possible outbreak scenario, allowing for generalizable insights into the effectiveness of interventions to delay a region’s first case. The model sheds light on how the effectiveness of lockdowns to slow the spread of disease is influenced by the interaction of mobility networks and socio-economic levels. We find that a negative relationship emerges between network centrality and the infection delay after a lockdown, irrespective of income. Furthermore, for regions across all income and centrality levels, outbreaks starting in less central locations were more effectively slowed by a lockdown. Using the Infection Delay Model, this paper identifies and quantifies a new dimension of disease risk faced by those most central in a mobility network.

## Introduction

Since the start of the COVID-19 pandemic, an active literature has evolved to study the spread and dynamics of the disease from mobility networks (Coelho et al. 2020; Peixoto et al. 2020; Chang et al. 2020; Levin et al. 2021) or socio-spatial perspectives (Lee et al. 2021; Li et al. 2021; Cordes and Castro 2020). However, very few studies look at how *both* socio-economic conditions and network properties interact, and how they influence outbreaks together (Chang et al. 2020; Nande et al. 2021). Chang et al. (2020) create a mobility network from cell phone mobility data and model the spread of COVID-19, identifying the importance of ‘superspreader’ points and higher infection rates among disadvantaged racial and socio-demographic groups. Nande et al. (2021) explore how evictions influence the spread of COVID-19 in various simulation scenarios, considering different policy responses and highly local contact networks. Further, while extensive work has been done to model the spread of the virus and non-pharmaceutical intervention effectiveness in terms of cases, hospitalizations, and deaths (Li et al. 2021; Cordes and Castro 2020; Flaxman et al. 2020; Oraby et al. 2021; Oka et al. 2021; Meo et al. 2020) there is a lack of emphasis on the timing of case spread, and how interventions can delay a region’s first infection. Given the spatio-temporal granularity of cell phone mobility data capturing responses to lockdown policies, it is now possible to develop generalized, preventative methodologies which seek to further our understanding of disease vulnerability, and better prepare for novel outbreaks or variants.

This paper develops the Infection Delay Model (IDM), a novel effective distance-based methodology that can be used for assessing how lockdowns can delay a region’s first case and their intersection with socio-economic inequalities. The IDM captures the difference between disease arrival times with and without a lockdown, using a novel application of cell phone mobility data for effective distance research. To develop a forward-looking understanding of the impacts of interventions on the timing of disease spread, a use-case of the IDM is presented which considers the potential variability of future outbreak scenarios. Drawing from recent studies of network-driven contagion phenomena (Brockmann and Helbing 2014; Iannelli et al. 2017; Balcan et al. 2009), we simulate epidemics from every node in the transport network. By connecting those simulations with socio-economic data, generalizable insights are uncovered which can be applicable beyond the specific spreading patterns observed during COVID-19.

This paper uses the Metropolitan Region of São Paulo (MRSP) as a case study to apply the IDM. Given its unique position as an area of early disease introduction and high intrastate transmission, COVID-19 studies in the MRSP can help with preparation for future variants of COVID-19 or other pandemics (Coelho et al. 2020; Candido et al. 2020).

## Background

### Network-based analyses of COVID-19

One branch of literature on COVID-19 has focused on mobility networks to model the spread of the disease and assess the risks of cases and deaths. The data sources used to generate such networks range from domestic and international flight records (Coelho et al. 2020; Kuo and Chiu 2021), to cell phone mobility records and geo-located visits to places of interest (Peixoto et al. 2020; Chang et al. 2020; Nande et al. 2021; Ferreira et al. 2021). The varying spatio-temporal granularity of the data sources used in these analyses have led to diverse outputs to identify regions at risk and explore how non-pharmaceutical interventions (NPIs) such as lockdowns impact mobility and vulnerability, but also how that impact might be different across wealthier or poorer regions (Gozzi et al. 2021).

This area of literature uses transport flows to construct aggregated networks of population movement. Various methods have been implemented to study COVID-19 risks on these mobility networks. Effective distance-based studies calculate the ‘distance’ between two regions based on the degree of mobility flows between them—more connected regions are effectively ‘closer’ (Coelho et al. 2020). The effective distance of a region from an outbreak location has been shown to be predictive of infection arrival times (Brockmann and Helbing 2014; Iannelli et al. 2017). Other studies build compartmental models on top of the mobility networks, calibrated to regional epidemic trajectories, and use epidemiological parameters and outbreak locations to simulate the course of an epidemic (Chang et al. 2020; Balcan et al. 2009; Peixoto et al. 2020; Nande et al. 2021). As greater mobility and person-to-person contact is associated with transmission, epidemic simulations can be run on mobility networks with adjusted levels of mobility or contact patterns to explore the impacts of real or hypothetical interventions on health outcomes (Levin et al. 2021; Nande et al. 2021).

### Socio-spatial analyses of COVID-19

A separate branch of literature on COVID-19 has focussed on disease vulnerability and its intersection with existing socio-spatial inequalities. The range of analyses includes studies on how socio-economic levels are associated with differences in terms of cases, hospitalizations, and deaths (Li et al. 2021; Cordes and Castro 2020), health care facility access (Pereira et al. 2021; Tao et al. 2020), and inequalities in NPI adherence (Li et al. 2021; Lee et al. 2021; Jay et al. 2020; Heroy et al. 2022). It is worth noting that some of these analyses also study the impact of mobility restrictions, by either incorporating them as a proxy for the intensity of the economic downturn associated with the lockdown (Bonaccorsi et al. 2020), or identifying likely determinants of spatial variations of reductions in mobility (Gauvin et al. 2021). Overall, these spatial analyses often seek to uncover how variables such as race and income relate to COVID-19 risks, to identify how existing inequalities are being compounded by the ongoing pandemic.

In an analysis of hospitalization and deaths in São Paulo, it was found that black and *pardo* Brazilians were more likely to be hospitalized and die of COVID-19 (Li et al. 2021). Similarly, an analysis of clusters and contextual factors of COVID-19 in New York City found that regions with larger black populations without health insurance had higher positive testing rates (Cordes and Castro 2020). Cell phone mobility data has also been used to study the interaction of lockdown adherence and socio-economic inequalities. Conceptualizing mobility restrictions as a luxury not everyone can afford, it has been found that more vulnerable individuals were less able to reduce their mobility—potentially due to a lower probability of furlough or teleworking opportunities (Lee et al. 2021; Li et al. 2021).

### Contributions

The first contribution of this paper is an integrated analysis of how the complex network properties of mobility patterns interact with socio-economic characteristics to produce disease risk. While the socio-spatial branch of literature has consistently identified intersections between socio-economic vulnerability and disease burdens (Li et al. 2021), current network-based studies either use socio-economic data to contextualize network-based results (Coelho et al. 2020; Gauvin et al. 2021), or include it to identify relationships between mobility reductions and socio-economic vulnerability (Pullano et al. 2020; Valdano et al. 2021; Gozzi et al. 2021). There is a lack of investigation into the interaction between network properties and socio-economic factors, and how they jointly drive the distribution of disease risk. It cannot be assumed that features such as network centrality and income are proxies for each other, justifying an investigation which explicitly examines both.

The second contribution of this study is the introduction of a new method that estimates the extent to which lockdown measures can slow down the spread of diseases while taking into account the spatial and temporal heterogeneity of disease spread in a network science approach. Existing network-based and socio-spatial research on cases, hospitalizations, and deaths fail to measure a crucial goal of early lockdowns, namely delaying the time until a region’s first case. Delaying disease onset with early interventions can buy time for health systems to increase hospital and intensive care capacity, and establish rapid testing sites (Rocha et al. 2021). To investigate this dimension of disease risk, we introduce the Infection Delay Model, an effective distance-based method of calculating disease arrival times under baseline and lockdown mobility scenarios. Current literature which explores rankings of disease arrivals using effective distances does so while assuming a single known outbreak location (Brockmann and Helbing 2014; Iannelli et al. 2017), or including a small subset of potential outbreak locations (Coelho et al. 2020). These studies also overlook how rankings of disease arrivals are shaped by socio-economic inequalities. Given recent literature on the outsized influence of the outbreak region on the trajectory of a communicable disease (Schlosser and Brockmann 2021), this study simulates outbreaks beginning in every region of the MRSP, to allow for generalizable findings that do not assume that the next outbreak will begin in the same region as the last.

## Methodology

### Data

#### Cell phone mobility data

**Fig. 1:**
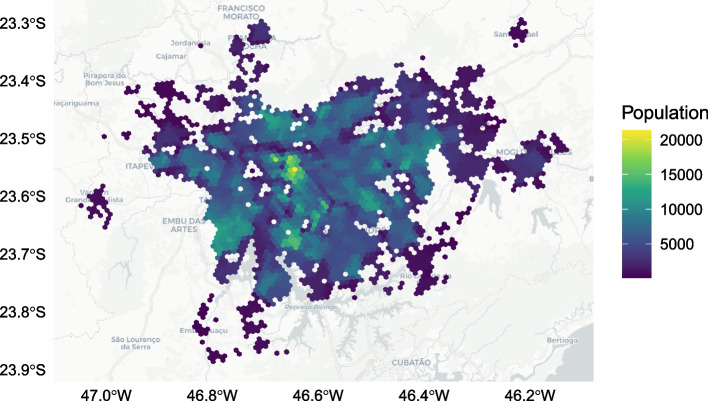
Geographic distribution of all hexagonal cells in the Metropolitan Region of São Paulo for which daily isolation levels are available from the mobile analytics company InLoco/Incognia (Incognia 2020). Shows variation in populations across the Metropolitan Region of São Paulo

Through an agreement with InLoco (Incognia 2020), a Brazilian cell phone analytics company now known as Incognia, this paper had access to daily isolation levels for MRSP from March 1, 2020 to April 19, 2020. These data come spatially aggregated on a hexagonal grid using the H3 index at resolution 8 (Brodsky 2018). The data set contains 2893 hexagonal cells of roughly 740 m$^2$ across the MRSP, of which 2599 had suitable time frames and auxiliary data after interpolation to be used in the analysis. The hexagonal isolation data is openly available in a data repository (see Availability of Data and Materials section). InLoco/Incognia gathers data by partnering with mobile phone applications, and uses software development kits to harvest location data while individuals are using partnered apps (Peixoto et al. 2020). This form of location gathering provides precise geo-coordinates, which are anonymized and aggregated to develop the social isolation indices. For a given hexagon cell, the proportion of individuals who reside in the cell and *stay* within it on a given day is recorded. This proportional value is used as a proxy for social isolation (Li et al. 2021), recording the extent to which individuals travel outside their residence area. Higher or lower social isolation values indicate that *fewer* or *more* individuals are leaving their residence area, respectively (Ferreira et al. 2021). The distribution of social isolation hexagon cells is presented in Fig. 1. The same data set was used in Li et al. (2021), showing that lower income individuals were less able to reduce their mobility after São Paulo’s lockdown, justifying our use of the cell phone data as capturing income-related mobility inequalities. The uneven coverage of the MRSP hexagon cells is a feature of the data set provided by InLoco/Incognia, discussed in the limitations section.

#### Travel survey data

The travel survey data for the MRSP were gathered from the 2017 MRSP household travel survey, conducted by the São Paulo Metropolitan Transportation Department between June 2017 and October 2018 (METRÔ-SP 2018). The original data set is a table of survey responses regarding the total daily trips of 86,318 individuals who reside in the MRSP. On average, each individual reports 2.12 daily journeys, leading to a total of 182,994 trip reports (METRÔ-SP 2018). Key information for the reports are the journey origin and destination, along with the travel time. The interviews were conducted across 39 municipalities within the MRSP, divided into 510 research zones for the purposes of the survey. Of all the research zones, 66% lie within the main municipality in the MRSP, São Paulo. The survey was designed to be statistically representative across the MRSP, and includes journey and population weights to scale responses by their frequency in the true population. From these weights, the total 2017 mobility flows between travel survey zones and 2017 estimates of populations were calculated. Population levels in 2020 were estimated by determining the geometric growth rate from 2010 and 2019 population totals, and scaling the 2017 populations to obtain population estimates per zone (Instituto Brasileiro de Geografia e Estatística 2010, 2019). Population counts for the commuting areas are necessary as inputs to the network-based compartmental epidemiological model used to simulate epidemics.

#### Census data

This study uses socio-economic data from the official 2010 Brazilian Census, focused on the census tracts within the MRSP (Instituto Brasileiro de Geografia e Estatística 2010; Pereira and Gonçalves 2019). Within the state of São Paulo, there are 68,296 tracts, with data included on the total population, racial aggregates, average income per capita (Brazilian Real per calendar month), functioning water networks, and other relevant socio-economic features. The census tracts within São Paulo state cover a larger area than both the cell phone mobility hexagon cells and travel survey zones, which are primarily focused on the MRSP. The population data from the the MRSP travel survey is more up to date than the 2010 census, therefore it is used in favour of the census data population totals. The census data remains useful for calculating regional income per capita averages, which are interpolated from census tracts into the hexagon cells.

#### Interpolating data to hexagon-level

While the social isolation hexagon cells provide spatially and temporally granular information on the daily proportion of residents leaving a given area, information on which population subgroups are included in each hexagon cell remain unknown. This problem is shared across the growing body of literature using cell phone mobility data for public health purposes, where anonymity measures by cell phone data providers obscure information on the sample (Grantz et al. 2020). While fundamental selection biases in the mobile phone data are a persistent issue, discussed in the limitations section, traditional data sources can be leveraged to generate population estimates within the hexagons (Aleta et al. 2020).

**Fig. 2:**
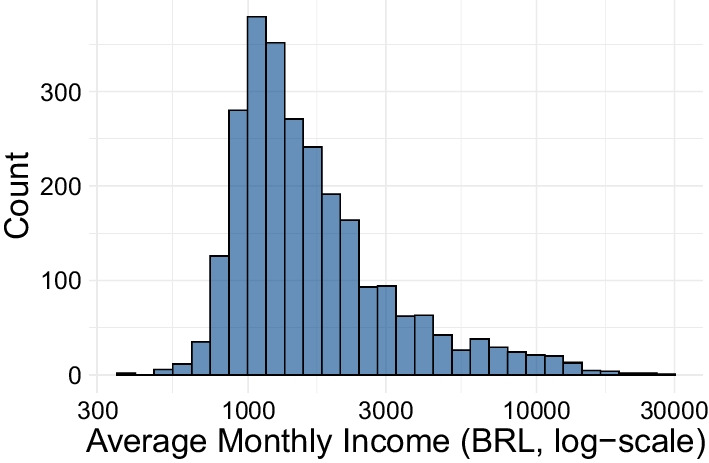
Distribution of average income per capita (Brazilian Real per month) across hexagon cells

The census tracts and travel survey zones are constructed of varying spatial structures which must be mapped to the social isolation hexagon cells. This process, known as spatial interpolation, is used in geospatial studies to estimate values in unknown area units using values in known geographic units (Comber and Zeng 2019). The spatial interpolation method used in this analysis is known as aerial weighting, which integrates socio-economic estimates based on proportional overlap (Comber and Zeng 2019). This method depends on the assumption of homogeneously distributed characteristics within census tracts and travel survey zones, but benefits from transparency and simplicity relative to interpolation methods which rely on auxiliary information (Comber and Zeng 2019). Each hexagon cell’s overlap with census tracts and travel survey zones was determined relative to their total areas. This proportional overlap area was used to generate a weighted allocation for income and population levels. For example, if a hexagon cell covered 50% of a travel survey zone with a population of 20, the hexagon cell would be assigned 10 individuals. Figure 1 geographically displays the interpolated populations across the hexagon cells in the MRSP, and Fig. 2 and Table 1 display the distribution of incomes.

**Table 1:** Summary statistics of average income per capita (Brazilian Real per month) across hexagon cells

	Monthly income (BRL/capita)
Mean	2172.079
Std	2318.79
Min	0.00
25%	1069.62
50%	1418.27
75%	2187.37
Max	28471.98

**Fig. 3:**
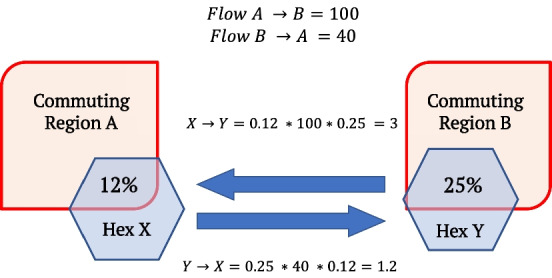
Travel survey data interpolation strategy from travel zones to hexagon cells. Outflow and inflow are proportional to a hexagon cell’s overlap with a travel zone. To estimate travel patterns within a given hexagon, known inflow and outflow between travels zones A and B are proportionally allocated based on the overlap. For example, Hexagon X overlaps with 12% of Region A, and therefore 12% of Region A’s *outflow* is assigned to Hexagon X. Hexagon Y overlaps with 25% of Region B, and therefore 25% of the 12% outflow is assigned to Hexagon Y as *inflow*

To interpolate the 2017 travel survey network to the hexagonal cells, the homogeneity assumptions of aerial weighting are extended to mobility flows between travel survey zones (Jang and Yao 2011). It is assumed that a hexagon cell overlapping with a given origin zone has a proportional quantity of outflow to all its targets. Similarly, hexagon cells overlapping with a given destination zone receive inflow from all relevant origin zones proportional to their intersection with that destination zone. An illustration of the travel flow interpolation to hexagon cells is provided in Fig. 3.

**Fig. 4:**
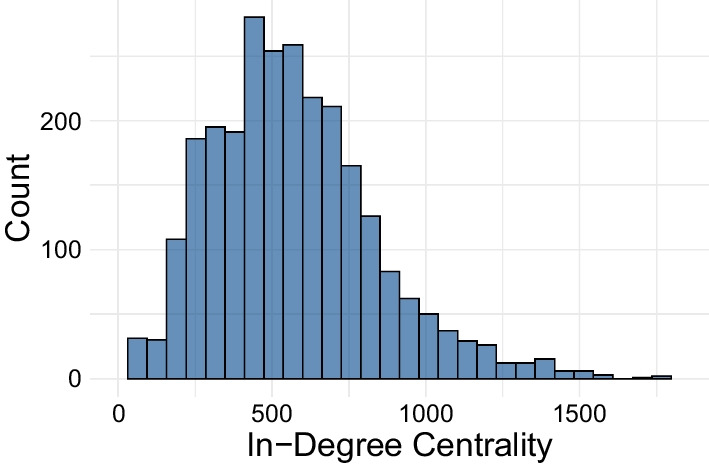
Distribution of in-degree centrality for hexagon cells in the interpolated mobility network

Based on the interpolated mobility network, the in-degree centrality of each hexagon cell is calculated. In-degree centrality is the number of edges that directly flow into a cell, representing the diversity of inflow connections—associated with a region’s time to infection (Hunter et al. 2020; Christley et al. 2005). The weighted in-degree (total travellers in) and weighted out-degree (total travellers out) are also highly correlated with the in-degree, and have been shown to influence the spread of disease (Francetic and Munford 2021). Other centrality measures to determine a node’s level of influence in a network include betweenness centrality, which measures the number of shortest paths that pass through a given node, and closeness centrality, which is the inverse of the geodesic distance from a given node to all others (Lü et al. 2016). Other, more recent measures focus on community structures and distinguish inter versus intra-community links when considering centrality (Rajeh et al. 2022). This includes the neighbourhood-based bridge node centrality, which measures how a node’s neighbours will belong to other network components if it is removed (Meghanathan 2021). In-degree is chosen in this analysis because the infection delay model focuses solely on initial disease *arrivals*, and in-degree centrality isolates these inward flows in a simple and interpretable measurement. The distribution of in-degree centrality in the hexagon-interpolated mobility network is presented in Fig. 4 and Table 2.

**Table 2:** Summary statistics of in-degree centrality for hexagon cells in the interpolated mobility network

	In-degree centrality
Mean	572.4
Std	268.6
Min	83.0
25%	383.0
50%	544.0
75%	719.0
Max	1829.0

### Infection delay model

This section will discuss in detail the methodology of the proposed Infection Delay Model. To provide an overview, the inputs to the Infection Delay Model are the effective distances from all pairs of hexagon cells, calculated under baseline and intervention mobility scenarios—the baseline being the scenario with no mobility restrictions, i.e. no form of lockdown. These effective distances are translated into two sets of infection arrival times, whose differences represent the ‘infection delay’ of an intervention. For a given outbreak location, the infection delay algorithm first determines how much time would be ‘added’ until every region’s first case if the outbreak location implemented a lockdown on the first day. Assuming a lockdown is not immediately implemented at the outbreak location, we simulate the unmitigated spread of the disease using a SIR model and calculate the infection delay of a lockdown intervention at every subsequent day based on the currently infected regions. This produces a time series plot for every region, known as its infection delay curve, showing the infection delay values over time for an outbreak beginning at a known region. While this provides estimates of the ‘time added’ to a region’s first case from a known location, we calculate and characterize infection delay curves for every region under every possible outbreak scenario to understand general trends.

#### Calculating effective distance

To calculate the effective distances for the hexagon-scaled mobility network, this analysis uses the ‘dominant path’ effective distance, a metric used in numerous disease arrival time analyses (Iannelli et al. 2017; Brockmann and Helbing 2014; Coelho et al. 2020; Gautreau et al. 2008), translated into Python by Iannelli et al. (2017). Measures of the dominant path effective distance focus solely on the most probable path of transmission from hexagon *i* to *j*. To calculate this value, for every connected origin *i* and destination *j* in the network, we calculate the transition rate as the proportion of total travellers beginning in hexagon *i* who arrive in hexagon *j*, denoted as $0 \le P_{ij} \le 1$ (Brockmann and Helbing 2014). The effective distance between hexagons *i* and *j* is calculated as:

1 $$\begin{aligned} d_{ij} = 1-\ln ({P_{ij}}), \end{aligned}$$

which is used as an edge weight for every pair of *i*, *j* hexagons, or nodes in the mobility network (Brockmann and Helbing 2014). These edge weights, greater than or equal to one, are used in a weighted shortest path analysis to determine the dominant path effective distance between every pair of hexagon cells. With $d_{ij}$ calculated for all edges in the network, the dominant path between *i* and *j* is chosen as the path which minimizes the sum of effective distance edge weights between them. Finally, the dominant path effective distance between two hexagons ($D_{ij}$) is calculated as the sum of the effective distances along the determined shortest path. This basic dominant path effective distance can be used to detect rankings of arrival times for a given outbreak location (Brockmann and Helbing 2014).

The traditional dominant path effective distance model is solely based on the mobility network, captured by $P_{ij}$, and does not have parameters which can incorporate changing epidemiological parameters or rates of mobility reduction in the network. To add epidemiological and mobility-based parameters, useful for a comparative analysis, the effective distance formula is altered to

2 $$\begin{aligned} d_{ij}= \left( \ln \left( \frac{\beta -\mu }{\kappa }\right) - \lambda \right) -\ln (P_{ij}), \end{aligned}$$

shown in Iannelli et al. (2017), where $\beta$ and $\mu$ are the infection and recovery rate. The mobility compound parameter $\kappa$, representing the proportion of the circulating population, is altered to incorporate mobility reductions, given by $(1.0-\textit{mobility reduction}/100.0)\times \kappa _0$, where the *mobility reduction* goes from 0 to $100\%$. In this compound parameter, $\kappa _0$ is the mobility rate, chosen to be 10%, which also ensures the logarithm is positive after the subtraction of $\lambda$, the Euler-Mascheroni constant (Iannelli et al. 2017). As $\kappa _0$ is constant between the baseline and intervention scenarios, its value does not impact the infection delay value when the differences in arrival times are calculated between the two. The reproductive number $R_0$ is chosen to be 2.9, based on an epidemiological characterisation of the MRSP early in the pandemic (de Souza et al. 2020). The infectious period is chosen to be 9.2 from a mathematical analysis of COVID-19 in Brazil (Pinto Neto et al. 2021). The infection rate is thus $R_0/\textit{infectious period}$ = 2.9/9.2, and the recovery rate is given by $1/\textit{infectious period}$=1/9.2. It is important to note that the transition rate $P_{ij}$ calculation is unaltered from the traditional model. As the mobility compound parameter $\kappa$ rises, $d_{ij}$ decreases, indicating that *i* and *j* are effectively closer. Similarly to the traditional model, for every potential outbreak and target hexagon cell in the network, the dominant path effective distance is generated from the weighted shortest path analysis, generating a $2599 \times 2599$ matrix of effective distances. This method is able to calculate effective distances between hexagons irrespective of whether they are directly or indirectly connected.

Two $2599 \times 2599$ matrices of effective distances are calculated for every potential origin *i* and destination *j*, under the following mobility flow scenarios:

1. No mobility reduction (baseline scenario).
2. Reduction in mobility based on hexagonal isolation changes.

The first scenario assumes no interventions, where arrival times are calculated using the baseline travel pattern information ($\textit{mobility reduction}=0$). The second scenario assumes that hexagon cells reduce their mobility by the same amount as observed during the first wave of the pandemic, through leveraging the cell phone social isolation information. To determine the extent of the mobility reduction for each region, the marginal change in social isolation from pre-lockdown to post-lockdown is calculated. The initial isolation value for each hexagon is calculated as the mean across March 1 to March 15, the two weeks leading up to the MRSP’s lockdown (Siciliano et al. 2020). The lockdown isolation value for each hexagon is calculated as mean from March 16 to March 30 2020, capturing the initial regional responses to lockdown measures. After determining the marginal change in real isolation for each origin hexagon, the effective distance calculation becomes:

3 $$\begin{aligned} d^{\textit{intervention}}_{ij}=\ln {\left( \frac{\beta -\mu }{\kappa ^{\textit{mobility reduction}}_i} - \lambda \right) } -\ln {(P_{ij})} \end{aligned}$$

then used to calculate the dominant path effective distance between all *i*, *j* nodes.

This representation of effective distance is used to approximate how rapidly a disease would spread from hexagon *i* to *j* given the observed change in pandemic isolation for region *i*. The adjustment of the compound $\kappa$ term to $\kappa ^{\textit{mobility reduction}}_i$ is a novel contribution of the study, allowing the analysis to capture heterogeneous changes in mobility based on cell phone mobility data, known to intersect with socio-economic vulnerability in the MRSP (Li et al. 2021).

#### Infection delay of intervention

To generate an estimation of arrival times based on the effective distances, this paper employs the methods used in Iannelli et al. (2017), dividing the effective distance by the effective velocity, defined as $V^{EF} \approx \beta -\mu$, where $\beta$ is the infection rate and $\mu$ is the recovery rate. The arrival time for a disease to arrive from location *i* to location *j*, including both the dominant path effective distance $D_{ij}$ (sum of shortest effective distance path from *i* to *j*) and velocity is thus:

4 $$\begin{aligned} T_{ij} = \frac{D_{ij}}{V^{EF}} \end{aligned}$$

Having generated the arrival times under both scenarios for every *i*, *j* combination, the infection delay by an intervention for an introductory case arriving from origin *i* to destination *j* is calculated as:

5 $$\begin{aligned} ID_{ij} = T^{\textit{intervention}}_{ij} - T^{\textit{baseline}}_{ij} \end{aligned}$$

The infection delay ($ID_{ij}$) values are calculated for every pair of hexagon cells, generating a $2599 \times 2599$ matrix where each *i*, *j* value represents the additional time to a case arriving from *i* to *j* given a mobility reduction proportional to *i*’s real mobility change.

Using known changes in mobility to understand intervention effectiveness takes into account the inequality in regional responses, and allows intervention scenarios to mimic the real capacities of hexagon cells to isolate and adhere to policy guidelines. Having the arrival times in $T^{\textit{intervention}}_{ij}$ reflecting the real mobility changes allows for an infection delay analysis which better captures the lived experience of each of the 2599 hexagon cells in determining the relative benefits from early interventions.

From the MRSP’s first case of COVID-19 to its widespread presence, this analysis determines the time ‘added’ until a region’s first case (infection delay) by an intervention at every hypothetical time *t*, assuming no intervention before *t*. At time $t=0$, only the initial outbreak location $i_0$ has the disease, and each hexagon’s infection delay by an intervention is $ID^0_{ij_0}$, representing the change in intervention arrival time relative to the baseline arrival time from $i_0$ to *j*. For every $t \ge 1$, each hexagon cell’s infection delay value is determined based on the currently infected regions. To calculate this value, for every hexagon cell *j* and discrete time step *t*, the following algorithm is developed:

1. Determine all infected hexagon cells at time *t*.
2. Determine the infection delay of an intervention across all currently infected hexagon cells relative to destination *j*.
3. Select the minimum infection delay value.

**Fig. 5:**
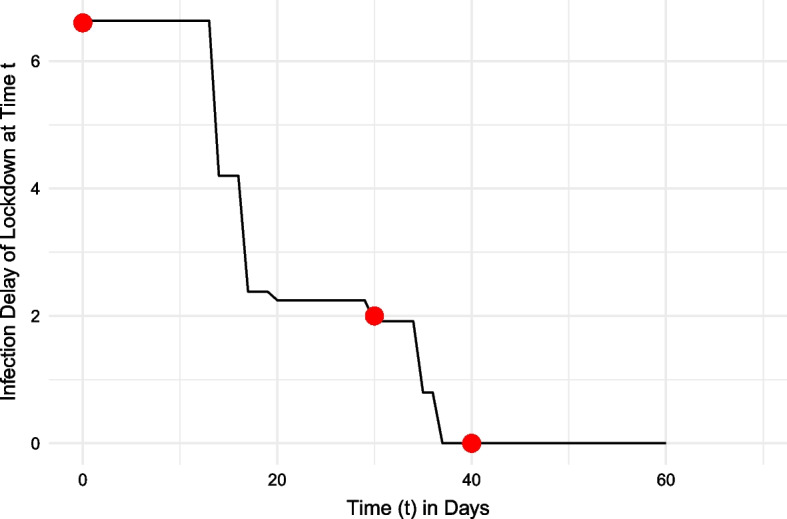
Hypothetical infection delay curve for region-at-risk *A* caused by a lockdown, following an outbreak beginning in region *B*. At time $t=0$, location *B* would be the only infected region—as the outbreak location. At this time, a lockdown would allow region *A* to gain approximately 6.6 days (y-axis) until its first case of COVID-19. If the disease were to spread unmitigated until time $t=30$ days, a lockdown would provide a gain of only 2 days before region *A*’s first case. At the 40-day mark following an outbreak in region *B*, without any intervention, region *A* would already be infected. Thus, a lockdown intervention at this point would have no ability to delay the onset of infection, with a y-axis value of 0

Following this algorithm, the IDM generates a time-series infection delay curve. An example plot is presented in Fig. 5, for a given hexagon *A* and outbreak location *B*. There are two primary factors that interact to create the structure of the infection delay curve: (1) the effective distance of infected hexagon cells to the hexagon cell of interest; (2) the degree of mobility reduction of infected hexagon cells. The outbreaks used in this analysis will be simulations calculated from a compartmental epidemiological model.

The example in Fig. 5 shows how the IDM can be used to estimate the time ‘added’ to all regions’ first cases in a scenario with a specific outbreak location *B*, known a priori. To generalize the findings of the infection delay analysis to outbreak scenarios other than those observed during COVID-19, epidemic outbreaks are simulated beginning in each of the 2599 hexagons in the MRSP. This paper uses a commuter susceptible-infected-removed (SIR) model to simulate the spread of the disease, where members of the population progress from susceptible, to infected, to removed ‘compartments’ (Salimipour et al. 2023). These models have been used in numerous studies with mobility networks to explore disease risk in relation to COVID-19 (Chang et al. 2020; Goel et al. 2021; Ajbar et al. 2021; Salimipour et al. 2023). This paper focuses on the initial outbreak of the disease in a short time interval, where SIR models have been shown as an effective predictor despite difficulty forecasting epidemic spread in the longer term (Moein et al. 2021). This paper employs the commuter-oriented susceptible-infected-removed (SIR) model used in Schlosser et al. (2021), on GitHub as *EpiCommute*. While the original model is used to simulate the spread of COVID-19 in 401 German counties, this analysis uses the 2599 social isolation hexagons, providing their interpolated populations and mobility flows.

For each outbreak scenario, the calculated arrival times are used in conjunction with the IDM to generate infection delay curves for every hexagon cell. The end result is 2598 infection delay curves for every hexagon (excluding its own outbreak), each one encapsulating the infection delay to the first case by an intervention at every time *t*.

#### Median infection delay values

**Fig. 6:**
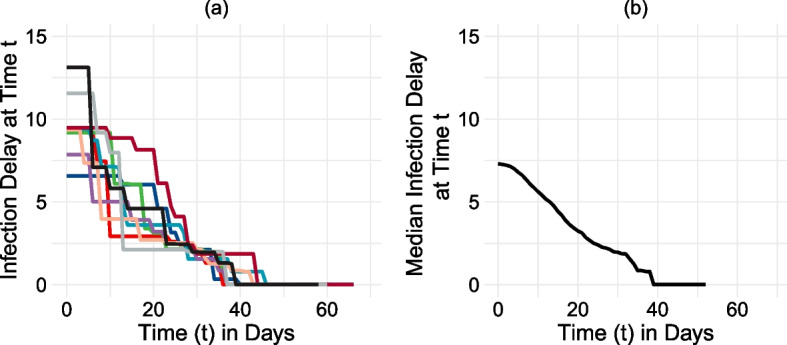
Illustrative example of infection delay median pipeline for a single hexagon cell, using only 10 outbreaks for visualisation (real analysis uses 2598 outbreak scenarios). From left to right: **a** the infection delay curves are calculated for each outbreak location; **b** the median of those curves are taken at every time *t* to create a general characterisation of lockdown effectiveness in the region-at-risk

To extract key information from each hexagon cell’s 2598 infection delay curves, the median value taken over the first 10 days is used to summarize the curve describing infection delay from an intervention. The first 10 days are chosen as they best exemplify the differences in infection delays across early outbreak scenarios, after which the curves begin to converge. Figure 6 displays the pipeline for calculating median infection delay curves for each hexagon cell. Rather than assigning every infection delay curve an equal weight and assuming that each scenario is equally likely, each curve is weighted by the in-degree centrality of its outbreak location, thus resulting in a weighted median value of the infection delay. In the first set of results, each hexagon cell is divided into centrality and income quartiles, and their relationships to infection delays are explored. A one-way ANOVA test is performed on the infection delay values to determine whether the differences are statistically significant. In the second set of results, each hexagon cell’s 2598 infection delay curves are divided into two groups based on the in-degree centrality of the outbreak location. A student’s t-test is performed on the two groups of infection delay values to test whether the differences are statistically significant.

## Results

### Weighted median infection delay curve

**Fig. 7:**
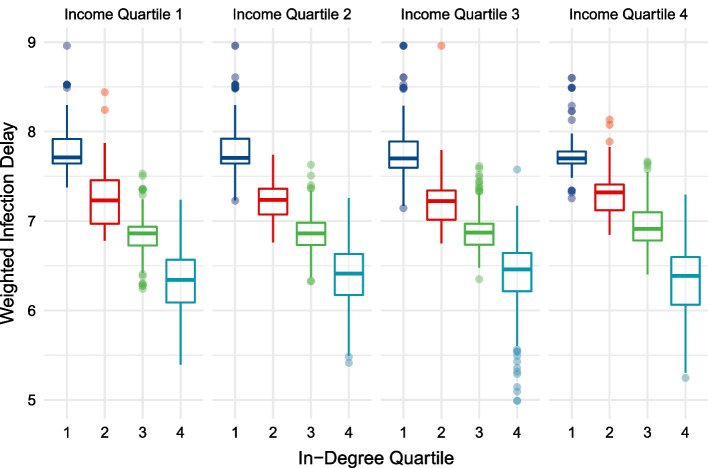
Weighted median infection delay values across in-degree centrality quartiles, while controlling for income

**Fig. 8:**
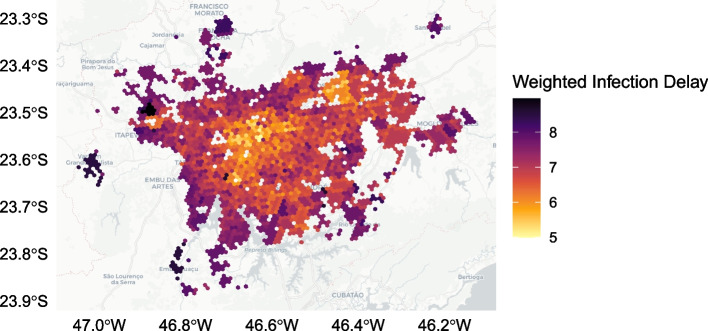
Geographic distribution of weighted median infection delay values over the first ten days of an outbreak

The relationship between greater centrality and lower infection delay values is displayed in Table 3 and Fig. 7. Within every income quartile, greater centrality is associated with a lower median infection delay value. These differences between infection delay values across centrality quartiles, controlling for income quartile, are statistically significant at the $p<0.01$ level based on the one-way ANOVA test. Figure 8 shows the geographic distribution of weighted median infection delay values.

**Table 3:** Weighted median infection delay values across income and in-degree quartiles

Income quartile (median BRL)	In-degree quartile (median in-degree)	Weighted median infection delay
1 (937.33)	1 (268)	7.71
	2 (452)	7.23
	3 (625)	6.86
	4 (813)	6.34
2 (1212.41)	1 (268)	7.70
	2 (468)	7.23
	3 (623)	6.86
	4 (791)	6.41
3 (1712.48)	1 (268)	7.70
	2 (470)	7.22
	3 (629)	6.87
	4 (847)	6.46
4 (3483.72)	1 (325)	7.70
	2 (469)	7.31
	3 (635)	6.91
	4 (944)	6.38

Median income per capita (Brazilian Real per Month) and in-degree centrality within each quartile subgroup is shown. The differences in weighted median infection delay values across in-degree centrality quartiles are statistically significant ($p<0.01$)

**Fig. 9:**
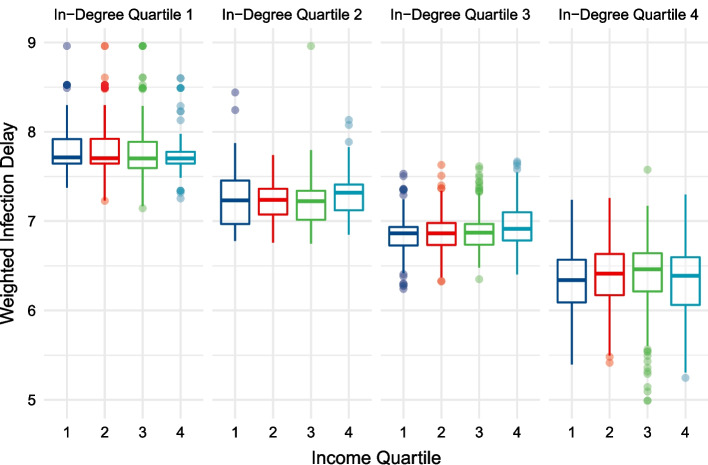
Weighted median infection delay values across income quartiles, while controlling for in-degree centrality

Figure 9 displays the distribution of infection delay curves across income groups, controlling for their levels of centrality. Observing the hexagon cells’ infection delays from Fig. 9, this analysis finds no discernable trend across income groups. The median infection delay values of hexagon cells in the bottom 25% of centrality are between 7.5 and 8 days. Hexagon cells in the highest centrality quartile all have median infection delay values between 6 and 6.5 days.

### Division by outbreak location centrality

**Fig. 10:**
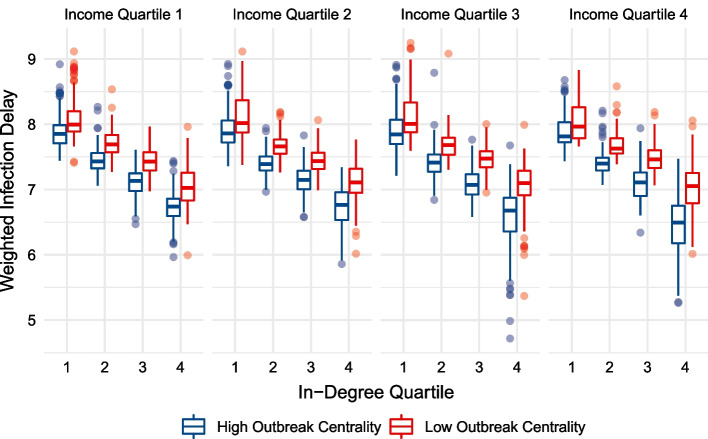
Un-weighted infection delay values across in-degree centrality quartiles, while controlling for income. Each region’s infection delay values are calculated and displayed for outbreak scenarios in the upper and lower 50% of in-degree centrality

**Fig. 11:**
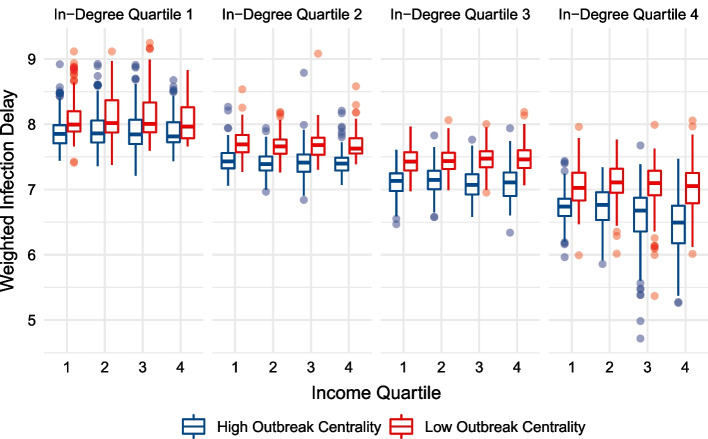
Un-weighted infection delay values across income quartiles, while controlling for in-degree centrality. Each region’s infection delay values are calculated and displayed for outbreak scenarios in the upper and lower 50% of in-degree centrality

Each hexagon cell’s infection delay value is subsequently calculated and shown when the outbreak location is in the bottom versus top 50% of centrality. For every hexagon cell, this creates two infection delay values, shown side-by-side in Figs. 10 and 11. We see that greater centrality is associated with lower infection delays, irrespective of income, and no clear pattern across income groups is observed when controlling for centrality—similarly to Figs. 7 and 9. The results also show that irrespective of the income and centrality grouping, outbreaks beginning in hexagon cells of lower centrality lead to greater infection delays of lockdowns. The student’s t-test indicates a statistically significant ($p<0.01$) difference between infection delay values depending on whether the outbreak location’s centrality is below of above the median.

## Discussion

This analysis has sought to uncover how the socio-economic and network characteristics of a region relate to the delay of its first case from an early intervention. The results of the Infection Delay Model indicate that the centrality of a region, independent of its income level, plays the largest role in determining how an early intervention will delay their first infection. There is no discernable relationship between income levels and the ability of a lockdown to slow the arrival of disease when controlling for centrality. This is surprising, considering that previous research using the same mobility dataset has shown that lower income individuals were less able to reduce their mobility after São Paulo’s lockdown (Li et al. 2021). Although previous studies have shown that vulnerable communities with lower isolation levels have higher infection rates of COVID-19 (Lee et al. 2021; Li et al. 2021; Cordes and Castro 2020), our results suggest that the influence of socio-economic and isolation inequalities in determining disease arrival is overridden by the outsized influence of centrality in the network. As an effective distance-based analysis, more central regions tend, on average, to be ‘closer’ to infected regions. This proximity reduces the potential infection delay of a lockdown, with an opposite mechanism in play for less central regions.

A potential reason why income does not have a clear impact on infection delay values, when controlling for centrality, is that socio-economic dynamics can be already embedded in the network topology. In Brazil, these dynamics have been shown to be at play, as lower-income regions face larger average commuting times (Pereira and Schwanen 2015)—a factor which would already be embedded in the commuter travel network used in this analysis. This study is not concluding that income does not have an effect on disease spread—priorly shown to do so in our region of study—but that in the IDM, income effects that are not already entangled with the network topology do not influence the delay to regions’ first cases caused by an intervention.

The literature produced during the COVID-19 pandemic has thoroughly highlighted the importance of socio-economic factors and their relationship to disease risk, rationalizing their use as more than a passive add-on to network-based results. The growing prioritization of socio-economic inequalities as a driving force of disease risk is exemplified in studies such as Nande et al. (2021), who study how eviction rates in Philadelphia have a measurable impact on the spread of COVID-19. The Infection Delay Model reflects socio-economic inequalities in the MRSP by incorporating real-life mobility reductions—known to be weaker in vulnerable areas (Li et al. 2021)—as a core component in the effective distance network analysis. Income is then used as a key axis to explore infection delays, found to be overpowered by a region’s centrality.

Rather than contradicting existing literature on the health burden inequalities associated with socio-economic status, this paper uncovers an unexplored perspective on pandemic preparedness. The emphasis of previous literature on case, death, and hospitalization counts illuminate how vulnerable groups are most at risk during the course of an outbreak (Li et al. 2021; Rocha et al. 2021; Jay et al. 2020; Lee et al. 2021; Cordes and Castro 2020; Pereira et al. 2021; Coelho et al. 2020). This paper targets a different, intervention-focused question: *How much time can be gained to a region’s first case from an early lockdown?* It cannot be assumed that the same mechanisms leading to greater disease risk *during* an outbreak lead to reduced intervention effectiveness *prior* to an outbreak. Our results, in conjunction with the established literature on socio-economic vulnerability and COVID-19, illuminate an additional burden faced by low-income, centrally located regions.

A major contribution of this study is its generalized, forward-looking characterisation of intervention effectiveness. Rather than relying on a single set of initial conditions when modelling a disease, or using a subset of transport hubs as outbreak locations, this analysis incorporates *all* possible outbreak locations when assessing how early interventions lead to infection delays. This allows for broad understandings of intervention effectiveness whose validity is not reliant on the next epidemic beginning in the same location as the last. This addresses the recently explored importance of outbreak locations on disease trajectories, providing generalizable insights for future disease preparedness (Schlosser et al. 2021). We are able to use the abundance of scenarios to generate weighted median infection delay values (Figs. 7, 9), emphasizing the dominant role of centrality. Further, we can divide outbreak locations into low and high centrality groups (Figs. 10, 11), and show that the infection delays of interventions vary based on the centrality of the outbreak location. We see that irrespective of the income or centrality quartile of recipient regions, outbreaks beginning in less central regions tend to lead to greater slowdowns.

### Conclusion

Research into the effectiveness of government interventions to slow disease spread is essential, as the disaster resulting from the COVID-19 pandemic and its emerging new variants continues globally. The novel Infection Delay Model proposed in this study provides a method of capturing how mobility reductions can slow the spread of an outbreak while considering the network patterns of mobility flows, an important element of intervention effectiveness. The data-linkage approach, interpolating travel behaviour and socio-economic data, allowed for insights into the social context of regions and how interventions can delay a region’s first case. The unique integration of cell phone mobility data into the effective distance metrics has captured heterogeneous changes in isolation, found in prior literature to intersect with socio-economic inequalities (Li et al. 2021; Lee et al. 2021). While this analysis is focused on Brazil, a region where income, health, and transport inequalities are stark (Malta et al. 2020), the presented approach can be applied in other regions to observe the intersection of intervention effectiveness, centrality, and socio-economic vulnerability. Similarly, the epidemiological parameters in this analysis are chosen to mimic COVID-19, but a novel variant or disease’s reproduction rate and infectious period could be used as substitutes. Adopting interdisciplinary methodologies to investigate the effectiveness of interventions, with a focus on exploring inequalities, may provide novel insights into the factors driving the unequal playing field exposed during the COVID-19 pandemic.

### Limitations and future directions

Based on the Infection Delay Model algorithm, the delays calculated for a given region are dependent on the reductions of mobility flows that arrive to it, rather than its own mobility reduction. This operates well under a regime where first cases arrive from individuals travelling from other locations. Advancements of the Infection Delay Model which capture how a first disease introduction to a region can originate from one of its residents travelling elsewhere would capture an important dimension of disease transmission. This may lead socio-economic and isolation inequalities to play a stronger role in shaping infection delay curves. Further, rather than calculating the time to a region’s first case, a case threshold such as 5% infection-rate of the population could be implemented, in which case a region’s own social isolation capabilities would more directly impact its infection delay value. These adaptations of the Infection Delay Model can expand its scope in capturing the concept of intervention effectiveness, as its current focus on the delay to a region’s first case is only one important element.

When considering cell phone data sources, originally collected for commercial purposes, coverage bias should be noted. As a cell phone analytics company, the sample of users in the Inloco/Incognia data set is determined by their market share, rather than an emphasis on representative samples (Tizzoni et al. 2014). The near-global ubiquity of cell phones does not preclude biases, as possession and use rates vary across demographic and income groups (Kraemer et al. 2020). The elderly are often underrepresented in such samples, while educated urban males are overrepresented relative to lower-income individuals (Kraemer et al. 2020).

In a preliminary analysis, a modified radiation model was used to determine if the results using real commuting data could be replicated with a generalized model. When observing the outbreak locations which led to above and below average infection delays for the rest of the MRSP, the radiation network overstated the influence of income-related mobility reductions relative to centrality. This may have occurred because the radiation model failed to replicate regional hubs with disproportionately large connectivity throughout the commuting network. This caveat should be considered for future research using effective distance-based metrics on artificially generated commuting data.

The suitability of integrating traditional household travel survey data with the aggregated social isolation cell phone data deserves exploration by future research. This study recommends comparing granular cell phone mobility location pairs, and observing how daily travel patterns and their changes after lockdown resemble those found in this paper’s analysis. If providing similar results, the greater anonymity of the aggregated social isolation data may render it a more readily accessible and minimally invasive method for granular mobility-related studies.

## Data Availability

The datasets and code supporting the conclusions of this article are both openly available. Data sets are stored as a Zenodo repository here: 10.5281/zenodo.5947174. The code is stored as a GitHub repository (Python/R) here: https://github.com/shivyucel/infection-delay-project, and archived at time of submission as a Zenodo repository here: 10.5281/zenodo.6008499.

## Abbreviations

NPI: Non-pharmaceutical intervention
MRSP: Metropolitan Region of São Paulo
IDM: Infection Delay Model
